# Amino Acid Profile and Mineral Content of Cultivated Snails *Acusta despecta* and *Achatina fulica*: Assessing Their Potential as Nutritional Source

**DOI:** 10.3390/foods14010123

**Published:** 2025-01-03

**Authors:** Sampat Ghosh, Min-Jung Kim, Sukjun Sun, Chuleui Jung

**Affiliations:** 1 Agriculture Science and Technology Research Institute, Andong National University, Andong 36729, Republic of Korea; sampatghosh.bee@gmail.com; 2 Forest Entomology and Pathology Division, National Institute of Forest Science, Seoul 02455, Republic of Korea; mjx10@korea.kr; 3 Department of Plant Medicals, Andong National University, Andong 36729, Republic of Korea; scv6309@naver.com

**Keywords:** edible snail, protein, minerals, iron, calcium, mini-livestock, nutrition security, heliciculture

## Abstract

This study evaluates the nutritional potential of two cultivated snail species, *Acusta despecta* and *Achatina fulica*, sourced from commercial farms in Korea, marking the first comprehensive analysis of *A. despecta*. The protein content of *A. despecta* (70.9 g/100 g dry matter) was significantly higher than that of *A. fulica* (44.2 g/100 g dry matter). Similar trends were observed for ash content (6.3 vs. 4.9 g/100 g dry matter) and crude fiber (2.9 vs. 0.4 g/100 g dry matter). Reflecting the higher protein content, *A. despecta* contained elevated levels of most amino acids compared to *A. fulica*. Glutamic acid was the most abundant amino acid, with leucine and lysine being the predominant essential amino acids in both species. The total amino acid content was 57.6 g/100 g dry matter for *A. despecta* and 40.4 g/100 g for *A. fulica*. Mineral analysis revealed significantly higher concentrations of minerals in *A. despecta*, except calcium and magnesium. Notably, *A. despecta* provided over 100% of the RDA/AI for calcium, phosphorus, copper, and manganese and met 94.5% of the iron RDA for women. These results emphasize the potential of *A. despecta* as a valuable dietary source for addressing protein and mineral deficiencies, particularly in nutrient-poor diets.

## 1. Introduction

The increasing global population has heightened the demand for food, posing a significant challenge to meeting nutritional needs sustainably [1]. As this demand grows, it is crucial to seek environmentally friendly food sources that do not exacerbate greenhouse gas emissions or further strain ecosystems, particularly in the context of climate change. The solution lies not only in improving modern agricultural practices but also in revisiting traditional and indigenous foods that have been utilized for generations across various cultures [2]. Among these underexplored food sources, snails stand out for their widespread acceptance as a dietary component in many regions [3]. Around the world, different communities have incorporated snails into their cuisines, recognizing them not only for their nutritional value but also for their medicinal properties [3,4,5,6]. However, while some cultures attribute health benefits to snail consumption, scientific research on their nutritional and bio-functional potential remains limited and warrants greater focus.

In South Korea, snail farming has become an established practice, primarily involving the rearing of different snail species for consumption and mucin (key component of snail mucus) extraction. This practice not only supports food diversity but also contributes to the development of snail-based products [7,8]. In previous studies, we have assessed the nutritional profile of *Pomacea canaliculata* [9] and detailed the farming methodologies associated with its cultivation [10]. Such research emphasizes the importance of understanding the nutritional composition of farmed snails, which could serve as a sustainable and protein-rich food source.

Evaluating the nutritional content of cultivated snails is vital for several reasons. First, it offers insight into their potential as an alternative protein source, aiding in food security and providing a sustainable dietary option with a lower environmental impact than conventional livestock [11,12]. Second, snail farming could foster economic growth by creating livelihood opportunities, particularly in underdeveloped and developing regions and where traditional agriculture faces climate-related challenges [13,14,15]. If proven to have high nutritional value, cultivated snails could be processed into various products with significant nutritional and functional benefits, further diversifying their use in the food industry.

This study focuses on the comprehensive nutritional analysis of two cultivated snail species, *Achatina fulica* (Achatinidae) and *Acusta despecta* (Camaenidae). *A. fulica*, commonly known as the African giant snail, is an edible species that is widely consumed across different countries of Africa and parts of Asia [16,17]. In many African countries, smallholder farms cultivate edible snails including this species, contributing not only to local food security by providing a nutritious protein source but also supporting the livelihoods of farmers engaged in snail farming [18]. This practice serves as an important economic activity, offering a sustainable means of income for communities. However, while the concept of snail farming is not new to Asia and some countries in the region do have snail farms, information on snail farming remains scarce, and research initiatives in this area are limited. Conversely, the use of *A. despecta* as a food source is less well documented, and there is a significant lack of data regarding its nutritional properties. Ethnozoological research on Jeju Island, however, has indicated that the local population consumes a soup made from this snail as a traditional remedy for a specific ailment for centipede bite [19]. *A. despecta* is commonly reared as pet in Korea and is being utilized for firefly rearing. Despite these cultural insights, the scientific literature detailing the nutrient composition and broader nutritional value of *A. despecta* is scarce.

This study aims to fill this gap by providing a comprehensive analysis of the nutritional profile of these two cultivated snails—*A. fulica* and *A. despecta*—from commercial farms. This study is especially significant for *A. despecta*, potentially offering the first in-depth data on its nutrient composition. The findings could highlight its potential as a valuable food source, supporting both dietary diversity and the development of alternative food options in sustainable nutrition. By assessing their macronutrient composition, amino acid profile, and minerals, we aim to position these species as potential candidates for sustainable food production. Additionally, the findings could guide the development of new food products that capitalize on the nutritional benefits of these snails, promoting both public health and local economic resilience.

## 2. Materials and Methods

### 2.1. Sample Collection and Preparation for Nutrient Analyses

**Figure 1:**
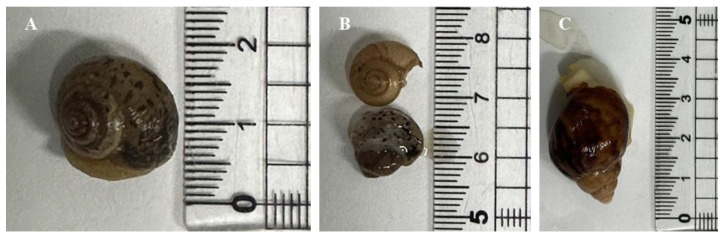
Specimens of *Acusta despecta* and *Achatina fulica*. (**A**) *A. despecta*; (**B**) *A. despecta* without the snail (bottom) and shell of *A. despecta* (top); (**C**) *Achatina fulica*.

The specimens of *A. despecta* and *A. fulica* used in this study were sourced from two separate commercial snail farms. *A. despecta* was collected from Snail Forest farm in Ulsan, which primarily breed snails for sale as pets and for educational purposes. *A. fulica* was collected from Yangpyeong Snail Farm in Yangpyeong, South Korea. Both species are land snails and are farmed in controlled environments, specifically in closed greenhouses within plastic pens, where temperature and humidity are carefully regulated. Snails are typically cultivated at temperatures ranging from 25 to 30 °C and humidity levels of around 80–90%. Generally, the snails are fed a diet consisting of rice germ, commercial swine feed, calcium powder, and soybean chunks. Upon collection, the snails were transported to the laboratory, where they were stored at −20 °C for preservation. The specimen of *A. despecta* measured approximately 1.5 cm in length, while *A. fulica* was 3 cm in length, as shown in Figure 1. This suggests that both snails were adults at the time of collection, with *A. despecta* being just over 6 months old and *A. fulica* around 2 months old.

The shells of the snails were removed, and the specimens were thoroughly cleaned under running water. After cleaning, the snails were dried with tissue paper to remove excess moisture, and then freeze-dried (Christ Alpha 1-4, Christ, Osterode am Harz, Germany) at −50 °C for a minimum of 96 h. Once dried, the samples were ground into a fine powder, which was subsequently stored at −20 °C until further chemical analysis.

### 2.2. Proximate Nutrient Composition Analysis

The proximate nutrient composition, including moisture, crude protein, crude fiber, and ash content, was analyzed using standard methodologies recommended by AOAC [20]. Moisture content was determined on a fresh weight basis, while other components were assessed on a dry matter basis. Crude protein was estimated using the Kjeldahl method with a Kjeldahl apparatus (SpeedDigester K-436 and Kjel Line distillation unit, Buchi, Switzerland) by measuring the nitrogen content and converting it to protein using a nitrogen-to-protein conversion factor of 6.25. Crude fat was determined using a Soxhlet apparatus (assembled glass wares and heating mantle of Daihan Scientific, Wonju, Republic of Korea) with petroleum ether as the solvent. Crude fiber was determined through acid and alkali digestion, and ash content was measured using a muffle furnace (Hanyang Science Lab, Seoul, Republic of Korea). Nitrogen-Free Extract (NFE) was calculated by subtracting the sum of all proximate components, i.e., protein, fat, fiber, and ash, from 100.

### 2.3. Amino Acid Analysis

The composition of proteinogenic amino acids was determined using standard AOAC [20] methods. The sample was digested with 6 N HCl at 110 °C for 24 h. The digested sample was then reconstituted with lithium citrate buffer and analyzed using an amino acid analyzer (Sykam S633, Sykam, Eresing, Germany). Results were expressed as g per 100 g of snail (excluding the shell) on a dry weight basis.

### 2.4. Mineral Analysis

Nutritionally important minerals, including calcium (Ca), magnesium (Mg), sodium (Na), potassium (K), phosphorus (P), iron (Fe), zinc (Zn), manganese (Mn), and copper (Cu), were analyzed following the standard methods outlined in the Korean Food Standard Codex [21]. The sample was digested using a mixture of HCl and HNO_3_ acids, and the mineral content was determined using ICP-AES (Agilent 5110, Agilent, Santa Clara, CA, USA). Results were expressed as mg per 100 g of snail (excluding the shell) on a dry weight basis.

We further evaluated the percentage of the Recommended Dietary Allowance (RDA) or Adequate Intake (AI) of individual minerals for both women and men, based on the consumption of 100 g of dry matter from the two studied snail species. The RDA values for most minerals and AI values for potassium, sodium, and manganese were obtained from the Harvard T.H. Chan School of Public Health’s Nutrition Source [22]. For this analysis, the higher RDA or AI values for adults were used as a reference.

### 2.5. Statistical Analysis

Snails were collected at one time and analyses were conducted on a single batch. However, all the samples collected for nutrient analysis underwent composite sampling, ensuring that the results represented the population as a whole, albeit at the specific developmental stage of the snails. Proximate composition and amino acid analyses were performed in triplicate, while mineral analysis was carried out in duplicate. The results are presented as mean ± SD. To compare the means, a *t*-test was conducted using SPSS (Statistical Package for Social Sciences) 16.0 (International Business Machines Corporation (IBM), Armonk, NY, USA), with a significance level set at *p* < 0.05.

## 3. Results

### 3.1. Proximate Nutrient Composition

Table 1 presents the proximate composition of the snail species *Achatina fulica* and *Acusta despecta*. The protein content was notably higher in *A. despecta* compared to that of *A. fulica*. The crude fat content was significantly higher in *A. despecta* compared to *A.* fulica; however, the overall fat content was low in both snail species. Similarly, ash content was significantly greater in *A. despecta* than in *A. fulica*. This trend was also observed for crude fiber, with *A. despecta* showing higher values than *A. fulica*. Since all proximate components were higher in *A. despecta*, for this simple reason, NFE content was consequently higher in *A. fulica* compared to *A. despecta*.

### 3.2. Amino Acid Composition

Table 2 illustrates the amino acid composition, with a total of seventeen amino acids analyzed. Presumably due to acid hydrolysis, tryptophan was not detected, and methionine and cysteine were not fully quantified. Reflecting the trend observed in total protein content, the levels of most individual amino acids were higher in *A. despecta* compared to *A. fulica*. Among the essential amino acids, leucine was the most abundant, followed by lysine. Overall, glutamic acid was the predominant amino acid in both species. Although the absolute values varied, the overall pattern of amino acid composition was consistent for both snails. The total amino acid content was 57.6 g/100 g dry matter for *A. despecta* and 40.4 g/100 g dry matter for *A. fulica*.

**Figure 2:**
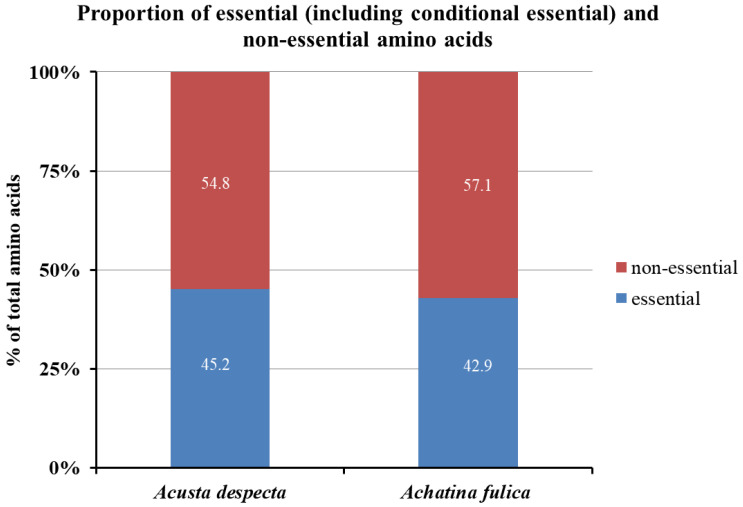
Proportion of essential (including the conditionally essential amino acid tyrosine) and non-essential amino acids in the total amino acid content of *Acusta despecta* and *Achatina fulica* (dry matter, without shell).

Based on the amino acid composition determined in this study, the proportion of essential and conditionally essential amino acids relative to the total amino acid content was calculated. For *A. despecta*, essential and conditionally essential amino acids made up 45.2% of the total amino acids, while for *A. fulica*, they accounted for 42.9%. These values, as illustrated in Figure 2, underscore the nutritional significance of both snail species. The high proportion of essential and conditionally essential amino acids highlights their potential as a valuable protein source, contributing to a balanced intake of amino acids essential for health and metabolic functions.

### 3.3. Mineral Content

The mineral content of the two snail species, *A. despecta* and *A. fulica*, is summarized in Table 3. All of the analyzed minerals, with the exception of calcium and magnesium, were observed to be present in significantly higher concentrations in *A. despecta* compared to *A. fulica*. This finding highlights the superior mineral profile of *A. despecta*, suggesting it as a more nutrient-rich option for dietary and nutritional applications.

Table 4 presents the percentage of RDA/AI fulfillment upon the consumption of 100 g of dry matter from *A. despecta* and *A. fulica*. Comparatively, *A. despecta* contained significantly higher levels of most minerals than *A. fulica*, resulting in greater RDA/AI satisfaction. Notably, calcium, phosphorus, copper, and manganese levels exceeded 100% of the RDA/AI with 100 g consumption of dry snails. *A. despecta* serves as a substantial dietary source of iron, meeting approximately 94.5% of the RDA for women.

## 4. Discussion

The proximate composition analysis, including crude protein, fat, fiber, and ash content, of the two snail species assessed in this study falls within the range reported for different edible snail species in previous research [3]. However, in this study, the crude protein content of *A. fulica* was found to be lower than the 62.6%, 63.1%, and 58.4% reported by Nkansah et al. [23], Etukudo et al. [24], and Tovignon et al. [25], respectively. Aboua [26] reported that the protein content of *Achatina fulica* was 72.1% in the flesh (excluding the shell) and 39.5% including the shell. The ash content of *A. fulica* was reported as 3% by Nkansah et al. [23] and 3.1% by Etukudo et al. [24], both of which are slightly lower than the value found in this study. Conversely, Nkansah et al. [23] reported an ash content of 0.3%, which is similar to the 0.4% observed in the present study. The variations in nutrient content can be attributed to factors such as diet [27,28], the developmental stages of the organism, and environmental conditions. Significantly, *A. despecta* exhibited a notable amount of crude fiber, which can offer dietary benefits when consumed as part of human nutrition. When evaluating food quality, protein content is regarded as one of the most essential components and ash content reflects mineral richness. The high protein and ash and lower fat content found in snails underscore their nutritional value, particularly in contrast to modern processed foods, which are often energy-dense and associated with adverse health outcomes such as obesity and cardiovascular diseases. Although the studied snails have a low crude fat content and fatty acid profiling remains a task for further research, it is generally recognized that a high proportion of unsaturated fatty acids, especially polyunsaturated fatty acids, in snail fat is nutritionally advantageous [3,9].

Unlike crude protein, which offers only an approximate measure, amino acid analysis confirms the presence of specific amino acids, which makes amino acid composition a more reliable indicator for understanding the nutritional value and characterizing the protein content of food. While the values for individual amino acids differ among various snail species, the overall amino acid composition patterns observed in both snail species studied in this research align with those reported in previous research on edible snails [3,9]. The amino acid profile further highlights the potential of these snails as a protein source. This composition is significant due to the critical functions of essential amino acids in human health.

Leucine, isoleucine, and valine are branched-chain amino acids (BCAAs), comprising about 17% of total amino acid present in human skeletal muscle [29]. They play critical roles in muscle protein synthesis, muscle metabolism and tissue repair, maintenance of nitrogen balance in the body, etc. Besides consuming foods enriched with BCAAs, BCAA supplements, especially leucine, are increasingly becoming popular, especially among athletes and clinical populations, to support muscle mass maintenance [30]. Given their high BCAA content, snails can serve as an excellent dietary source of BCAAs. Additionally, snail-derived proteins could be isolated and developed into valuable protein supplements. Lysine is frequently the limiting amino acid in cereals [31]. Consequently, cereal-based diets, which are common is least developed regions, may be deficient in lysine unless supplemented with lysine-rich dietary sources. The high proportion of lysine in the protein profile of edible snails, as observed in this study and corroborated by previous reports [3], enhances their nutritional value, positioning them as a valuable dietary supplement for improving overall protein intake. Methionine is often a limiting amino acid for protein synthesis and is typically deficient in plant-based proteins [32]. It plays a crucial role in the synthesis of cysteine through the trans-sulfuration pathway, which is a rate-limiting step in the production of glutathione, an important antioxidant molecule [33]. Threonine plays a role in protein synthesis, supports immune function, and aids in fat metabolism [34,35]. While the acid hydrolysis process has limitations in entirely estimating these amino acids, both methionine and cysteine are present in snail protein. Among the three aromatic amino acids (AAAs)—tryptophan, phenylalanine, and tyrosine—the first two are essential amino acids, while tyrosine is considered a conditional essential amino acid. Tyrosine, which is derived from phenylalanine, serves as a precursor for the biosynthesis of important molecules such as DOPA, dopamine, octopamine, norepinephrine, epinephrine, and melanin [36]. Histidine is crucial for growth, tissue repair, and the production of histamine, which is involved in immune responses and sleep regulation [37]. Tryptophan was not detected, possibly due to the limitations of the acid hydrolysis process; however, the other AAAs (tyrosine, phenylalanine), histidine, and threonine were found in significantly higher levels in snail protein. This suggests that incorporating snails into the diet can enhance protein and essential amino acid intake, particularly in regions where protein-deficient diets are prevalent.

Hidden hunger, characterized by deficiencies in essential micronutrients, is prevalent in many parts of the world, particularly in least developed and developing regions [38]. This condition is often linked to a lack of minerals such as calcium, iron, and zinc. Exploring the potential of snails as a dietary source of these minerals could be a valuable approach to addressing these deficiencies, as snails are generally rich in minerals.

The mineral content measured in the two studied snail species aligns with the ranges reported in previous studies for most minerals, though variations are species-specific [3]. The calcium content of *Achatina fulica* was found to be higher than the value reported by Aboua [26], amounting to 1060 mg per 100 g. Calcium plays a crucial role in bone and tooth formation, muscle contraction, nerve signaling, and blood clotting [39]. Dietary calcium deficiency is a global issue, affecting nearly half of the world’s population due to insufficient access to calcium-rich foods [40]. According to Shlisky et al. [40], populations in low- and middle-income countries (LMICs) are at the highest risk of inadequate calcium intake. However, a significant number of individuals in high-income countries (HICs) also fail to meet the recommended calcium intake levels. The condition of calcium deficiency is more pronounced in elderly populations, leading to conditions like osteoporosis [41]. Snails, being a rich source of calcium, could provide a significant portion of the required calcium intake. Assuming good bioavailability, their consumption may help mitigate the risk of calcium deficiency. Iron is another noteworthy mineral, as deficiencies are highly prevalent in the developing world [42] and are common among women, particularly those who are pregnant or of childbearing age, leading to iron-deficiency anemia [43]. Iron is essential for oxygen transport via hemoglobin and supports energy metabolism and immune function. *A. despecta* is particularly rich in iron, making it a promising dietary source to help combat iron-deficiency anemia.

Magnesium is critical for over 300 enzymatic reactions, including energy production, protein synthesis, and cardiovascular health [44]. Sodium and potassium help maintain fluid balance, nerve transmission, and muscle function, with potassium also regulating blood pressure and heart rhythm [45]. Zinc contributes to immune function, wound healing, and DNA synthesis, while manganese aids in bone formation, metabolism, and antioxidant defense [46,47,48]. Copper plays a role in iron metabolism, red blood cell production, and connective tissue development [39]. Both snail species are rich in minerals and meet a significant portion of the recommended dietary requirements, except for sodium and potassium. However, sodium intake is generally not a concern, as table salt typically provides an adequate amount.

While understanding their nutritional profile is essential, it is equally important to address safety concerns, particularly heavy metal contamination. Studies have highlighted the ability of snails to accumulate heavy metals and organic pollutants in their tissues [49,50,51]. However, both studied snail species were sourced from commercial snail farms and reared in enclosed rearing facilities, minimizing their exposure to environmental pollutants. Investigating potential heavy metal contamination remains an important area for future research.

## 5. Conclusions

Based on the findings of this study, *A. despecta* demonstrated superior nutritional potential compared to *A. fulica* in terms of protein content, amino acid composition, and the concentration of most minerals, except calcium and magnesium. The inclusion of snails in the diet could play a pivotal role in addressing protein and mineral deficiencies, particularly in populations dependent on nutrient-poor diets. As a rich source of high-quality protein, essential amino acids, and minerals such as calcium, iron, and zinc, snails hold promise for improving nutritional outcomes and overall health, especially in regions where dietary protein is scarce.

Furthermore, the high concentrations of BCAAs, lysine, calcium, and iron in snails suggest their potential for development into bio-functional components or fortifying agents when incorporated into other food products. This could significantly enhance the nutritional value of food systems. Additionally, promoting the farming of these snails as a form of “mini-livestock” could provide a sustainable and ecologically viable source of nutrition while simultaneously supporting nutrition security and boosting local livelihoods and economies. Future research should focus on the bioavailability of these nutrients and the development of snail-based food products to maximize their health benefits and commercial potential.

## Figures and Tables

**Table 1:** Moisture content (g/100 g fresh weight without shell) and proximate composition (g/100 g dry matter without shell) of *Acusta despecta* and *Achatina fulica*.

	*Acusta despecta*	*Achatina fulica*	*p*
Moisture	85.3 ± 0.55 ^a^	78.5 ± 0.56 ^b^	0.000
Crude protein	70.9 ± 0.73 ^a^	44.2 ± 0.49 ^b^	0.000
Crude fat	2.7 ± 0.21 ^a^	2.0 ± 0.26 ^b^	0.020
Crude ash	6.3 ± 0.04 ^a^	4.9 ± 0.06 ^b^	0.000
Crude fiber	2.9 ± 0.04 ^a^	0.4 ± 0.28 ^b^	0.004
NFE	19.7 ± 0.92 ^a^	46.0 ± 0.35 ^b^	0.000

Superscripts (^a,b^) indicate significant differences in nutrient content between two studied snail species; *p*-value of <0.05 is considered statistically significant.

**Table 2:** Amino acid composition (g/100 g dry matter without shell and % of total amino acid) of *Acusta despecta* and *Achatina fulica*.

Amino Acid	*Acusta despecta*		*Achatina fulica*		*p*
	g/100 g Dry Matter	%	g/100 g Dry Matter	%	
Aspartic acid	4.8 ± 0.05 ^a^	8.3	3.4 ± 0.13 ^b^	8.5	0.042
Threonine *	3.2 ± 0.03 ^a^	5.6	2.1 ± 0.08 ^b^	5.2	0.034
Serine	3.2 ± 0.04 ^a^	5.6	2.3 ± 0.04 ^b^	5.7	0.002
Glutamic acid	8.1 ± 0.01 ^a^	14.1	6.0 ± 0.30 ^a^	15.0	0.062
Proline	3.2 ± 0.08 ^a^	5.6	2.3 ± 0.13 ^b^	5.7	0.014
Glycine	3.5 ± 0.06 ^a^	6.1	2.7 ± 0.19 ^a^	6.7	0.109
Alanine	3.4 ± 0.02 ^a^	5.9	2.3 ± 0.11 ^b^	5.7	0.045
Valine *	3.2 ± 0.02 ^a^	5.6	2.1 ± 0.05 ^b^	5.2	0.025
Cysteine	0.6 ± 0.05 ^a^	1.0	0.4 ± 0.01 ^a^	1.0	0.120
Methionine *	0.8 ± 0.01 ^a^	1.4	0.7 ± 0.02 ^b^	1.7	0.014
Isoleucine *	2.8 ± 0.03 ^a^	4.9	1.8 ± 0.06 ^b^	4.5	0.002
Leucine *	5.1 ± 0.03 ^a^	8.9	3.4 ± 0.04 ^b^	8.5	0.000
Tyrosine **	2.3 ± 0.01 ^a^	4.0	1.5 ± 0.02 ^b^	3.7	0.000
Phenylalanine *	2.9 ± 0.03 ^a^	5.0	1.9 ± 0.09 ^b^	4.7	0.045
Histidine *	1.4 ± 0.01 ^a^	2.4	0.9 ± 0.00 ^b^	2.2	0.007
Lysine *	4.3 ± 0.02 ^a^	7.5	2.8 ± 0.08 ^b^	7.0	0.025
Arginine	4.7 ± 0.11 ^a^	8.2	3.5 ± 0.07 ^b^	8.7	0.006
Total	57.6 ± 0.08 ^a^	--	40.4 ± 1.30 ^b^	--	0.034

* indicates essential amino acid; ** indicates conditional essential amino acid. Superscripts (^a,b^) indicate significant differences in nutrient content between two studied snail species; *p*-value of <0.05 is considered statistically significant.

**Table 3:** Mineral content (mg/100 g dry matter without shell) of *Acusta despecta* and *Achatina fulica*.

	*Acusta despecta*	*Achatina fulica*	*p*
Ca	1282.6 ± 5.36 ^a^	2008.3 ± 0.15 ^b^	0.003
P	1251.2 ± 1.53 ^a^	699.2 ± 0.03 ^b^	0.001
Mg	327.3 ± 0.81 ^a^	343.0 ± 0.40 ^b^	0.026
Na	379.3 ± 0.79 ^a^	222.9 ± 0.12 ^b^	0.002
K	280.8 ± 1.13 ^a^	203.1 ± 0.24 ^b^	0.007
Fe	17.0 ± 0.89 ^a^	8.1 ± 0.29 ^b^	0.047
Zn	25.3 ± 0.08 ^a^	4.6 ± 0.02 ^b^	0.002
Cu	11.2 ± 0.03 ^a^	4.1 ± 0.03 ^b^	0.000
Mn	21.2 ± 0.03 ^a^	3.3 ± 0.00 ^b^	0.001

Superscripts (^a,b^) indicate significant differences in nutrient content between two studied snail species; *p*-value of <0.05 is considered statistically significant.

**Table 4:** Percentage (%) of RDA/AI satisfaction upon consumption of 100 g dry matter of *Acusta despecta* and *Achatina fulica*.

Minerals	RDA/AI (mg) *		% of RDA Satisfied Upon Consumption of 100 g (Dry Matter)			
			*Acusta despecta*		*Achatina fulica*	
	Women	Men	Women	Men	Women	Men
Ca	1200	1200	106.9	106.9	167.4	167.4
P	700	700	178.7	178.7	99.9	99.9
Mg	320	420	102.3	77.9	107.2	81.7
Na	1500 **	1500 **	25.3	25.3	14.9	14.9
K	2600 **	3400 **	10.8	8.3	7.8	6.0
Fe	18	8	94.5	212.5	45.1	101.5
Zn	8	11	315.9	229.8	57.1	41.5
Cu	0.9	0.9	1241.0	1241.0	451.7	451.7
Mn	1.8 **	2.3 **	1176.3	920.6	181.2	141.8

* RDA/AI values for minerals were obtained from the Nutrition Source, Harvard T.H. Chan School of Public Health [22]. Higher values were considered for adults; ** AI

## Data Availability

The original contributions presented in this study are included in the article. Further inquiries can be directed to the corresponding author.
